# Socio-economic demands and challenges for non-invasive disease diagnosis through a portable breathalyzer by the incorporation of 2D nanosheets and SMO nanocomposites

**DOI:** 10.1039/d1ra02554f

**Published:** 2021-06-15

**Authors:** Ramji Kalidoss, Radhakrishnan Kothalam, A. Manikandan, Saravana Kumar Jaganathan, Anish Khan, Abdullah M. Asiri

**Affiliations:** Department of Biomedical Engineering, Bharath Institute of Higher Education and Research Selaiyur Tamil Nadu 600 073 India ramji.sat@gmail.com +91-9840-959832; Department of Chemistry, College of Engineering and Technology, SRM Institute of Science and Technology Kattankulathur Tamil Nadu 603 203 India; Department of Chemistry, Bharath Institute of Higher Education and Research Selaiyur Tamil Nadu 600 073 India; Centre for Nanoscience and Nanotechnology, Bharath Institute of Higher Education and Research Selaiyur Tamil Nadu 600 073 India; Bionanotechnology Research Group, Ton Duc Thang University Ho Chi Minh City Vietnam; Faculty of Applied Sciences, Ton Duc Thang University Ho Chi Minh City Vietnam; Department of Engineering, Faculty of Science and Engineering, University of Hull HU6 7RX UK saravana@tdtu.edu.vn; Chemistry Department, Faculty of Science, King Abdulaziz University Jeddah 21589 Saudi Arabia; Center of Excellence for Advanced Materials Research, King Abdulaziz University Jeddah 21589 Saudi Arabia

## Abstract

Breath analysis for non-invasive clinical diagnostics and treatment progression has penetrated the research community owing to the technological developments in novel sensing nanomaterials. The trace level selective detection of volatile organic compounds (VOCs) in breath facilitates the study of physiological disorder and real-time health monitoring. This review focuses on advancements in chemiresistive gas sensor technology for biomarker detection associated with different diseases. Emphasis is placed on selective biomarker detection by semiconducting metal oxide (SMO) nanostructures, 2-dimensional nanomaterials (2DMs) and nanocomposites through various optimization strategies and sensing mechanisms. Their synergistic properties for incorporation in a portable breathalyzer have been elucidated. Furthermore, the socio-economic demands of a breathalyzer in terms of recent establishment of startups globally and challenges of a breathalyzer are critically reviewed. This initiative is aimed at highlighting the challenges and scope for improvement to realize a high performance chemiresistive gas sensor for non-invasive disease diagnosis.

## Abstract 2

Breath analysis for non-invasive clinical diagnostics and treatment progression has penetrated the research community owing to the technological developments in novel sensing nanomaterials.

## Graphical abstract

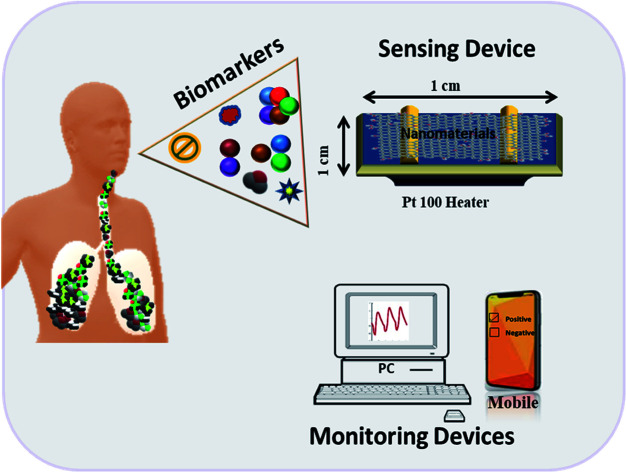

## 1. Introduction

Disease diagnosis through exhaled breath analysis has gained momentum during the past decade as the technology offers insights about the subject's internal body metabolism without the need for sample preparation. Among other non-invasive sources (tears, sweat, urine and feces) for disease diagnosis, exhaled breath analysis seems feasible as the ancient Greek physicians had predicted that the aroma of breath provides a certain clue about diagnosis.^1,2^ For instance, the smell of exhaled breath of patients with uncontrolled diabetes is often described as “rotten apple” due to the existence of acetone along with the mixture of inorganic vapors (*e.g.*, NO, CO_2_*etc.*), volatile organic compounds (*e.g.*, acetone, methyl nitrate, isoprene *etc.*) and other non-volatile vapors (*e.g.*, nitrogen, cytokines *etc.*).^3^

The advent of modern breath analysis came into existence when Pauling and his team in 1971 discovered more than 200 VOCs in human breath due to the biochemical pathways resulting through the physiological process.^4^ Usually, this mixture of vapors along with approximately 3500 chemical species existing in human exhaled breath have been identified by various analytical techniques including gas chromatography-mass spectrometry (GC-MS), flame ionization spectrometry (FIS) and photo ionization detection (PID).^5–11^ However, these methods are inadequate for routine breath biomarker monitoring as they rely on sophisticated laboratory equipment, trained technicians, and time consuming and complex sample preparation procedures leading to the lack of real time quantitative data. Thereby limiting the portability and so eliminates the possibility of point of care real time diagnosis. Hence the global scientific community had focused their efforts on developing a portable instrumentation for the real-time quantification of biomarkers for the disease diagnosis through exhaled breath.

The composition of VOCs range between parts per million (ppm) to parts per billion (ppb) that varies quantitatively and qualitatively for every individual similar to unique fingerprints.^12^ These VOCs may originate from cellular levels due to their fundamental body processes^13^ and metabolisms in blood^14^ that plays a vital role in altering the concentration of VOCs. Other sources include inhaled atmospheric air, airway surfaces and tissues throughout the body. As the blood collects all the compounds during bodily metabolism and interacts with lung, they appear in breath.^15^ 54 VOCs at elevated concentration indicates several health risks related to gastrointestinal, respiratory systems and metabolic disorders such as halitosis, renal failure, diabetes, chronic liver and kidney diseases few of them listed in Table 1.^31,32^ An increased concentration of ammonia is closely associated with renal disease, acetone with diabetes mellitus (DM) and nitric oxide with chronic obstructive pulmonary disease (COPD).^33^ Since all this VOC appear in the breath because of certain body metabolisms, these endogenous gases resolve practical information on the possible disease and hence it is the perfect indication of any disease. Accurate quantification of these biomarkers provides the feasibility of non-invasive disease detection through exhaled breath.

**Table 1:** List of breath biomarkers and their corresponding diseases

Biomarker	Disease	Ref.
Ethyl butanoate	COVID-19	16
Ammonia	Chronic kidney disease, liver dysfunction	17 and 18
H_2_S	Halitosis	19 and 20
NO	Asthma	21 and 22
HCHO, toluene, benzene	Lung cancer	23 and 24
Acetone	Type 1 diabetes	25 and 26
	Type 2 diabetes	
CH_4_	Intestinal anaerobes	27 and 28
Ethanol	Alcohol consumption	29 and 30

The availability of unlimited sample quantity even during unconsciousness may become vital in emergency situations, for continuous monitoring of disease progression and the effect of medication in short time period.^34,35^ Whereas, other non-invasive sources such as saliva, sweat, urine and feces need human intervention limiting the diagnostic capabilities at the situation of emergencies. While the biomarkers from each source originate from the fundamental body metabolism, exhaled breath is reliable as it eliminates the social awkwardness or an embarrassment to the patients during sample collection. Apart from social consideration, other non-invasive sources possess certain issues in terms of stability of the sample.^36^ Moreover with the capability of repeated and self sampling, breath analysis are considered to be truly non-invasive and can be performed easily without any embarrassment or discomfort.^37^ Also, it does not present burden to the subject being tested and the ease of sampling offers the advantage of delivering result on spot instead of the traditional laboratory sample preparation and analysis.^38^ Hence, the significance of breath analysis lies on eliminating the pre-analytical and post-analytical procedure along with the mailing of test reports to the concerned subject. Furthermore, as the biomarkers in breath appear due to the fundamental body metabolism at cellular level, diseases may be identified at early stage which is the need of the decade for different cancers that could be cured, if identified at preliminary stage.^39,40^ Breath analysis may permit bedside and personalized home care monitoring, thus could be inexpensive and allows frequent testing for proper control of the disease progression and the effect of medications.^41^ Thus offers an inexpensive replacement for the traditional laboratory analytical tests.

However, there are numerous challenges to be addressed to bring a portable breathalyzer to the commercial market. The high complexity of breath samples leads to misinterpretation of results.^42^ The exhaled biomarkers not only emanate from fundamental body processes but also from the exogenous production by different sources. Breath sampling procedure is not yet standardized leading to the unawareness of sample collection from either nasal or oral cavity. Even though, after the collection of idealized sample, extrinsic factors such as temperature and humidity may influence the outcome of the diagnosis. Furthermore, the level of data interpretation needs standardization to develop a cloud of breathprint database from laboratories across the globe representing a reference standard for clinical practices.^43^

Although, limited number of breathalyzer were recognized by international guidelines and used in patients. Among them ethanol breath test, nitric oxide breath test to diagnose asthma, urea breath test for the diagnosis of *Helicobacter pylori* and hydrogen breathalyzer for small intestine bacterial overgrowth are currently in research practices.^44–48^ Nevertheless, there is no single breath test act as stand-alone diagnostic test and only act as a pre-screening tool.

Incorporation of nanomaterials in gas sensors has attracted research interests owing to their unique physic-chemical properties. Gas sensor technologies are classified based on their differences in working principle and device structures. Broadly it is categorized into technologies that rely on the change in electrical and other properties including optical, calorimetric and acoustic properties. The reliability of technologies based on variations in electrical properties such as field effect transistors (FET), surface work function transistors (SWF) and chemiresistors are suitable for exact quantification of breath biomarkers in the range of parts per billion to parts per million. Among them, chemiresistors are widely explored due to their ease in fabrication and miniaturization, simplicity in operation and demands low power.^49^

Hence, the international breath research community is focusing on nanostructured chemi-resistive gas sensors for sensitive and selective detection of breath biomarkers. Herein, we will review SMO and 2DM nanocomposites in chemi-resistive gas sensing device configuration for the development of breathalyzers. Their corresponding sensing mechanisms and performance to various biomarkers was highlighted along with the social demands, challenges and regulatory aspects in the hope of directing future research towards non-invasive disease diagnosis through portable breathalyzers.

## 2. Chemi-resistors

**Fig. 1:**
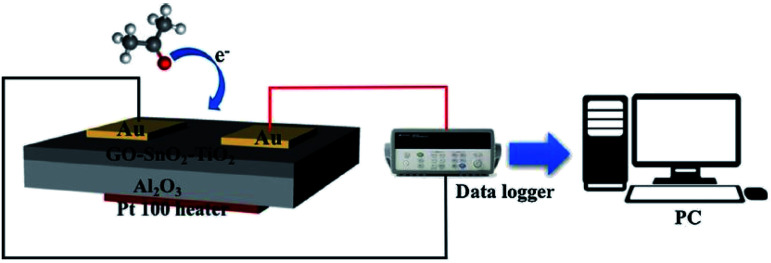
Schematic illustration of chemiresistor gas sensing device configuration reproduced from ref. 54 with permission from Elsevier, Copyright 2021.

Chemiresistors consists of a sensing film supported on an inert substrate where interdigitated or two metallic electrodes on their either side are deposited and a heater printed at the backside.^50^ This gas sensor device configuration was explored in sensing various biomarkers including acetone, nitrous oxide, ammonia *etc.* and put to use for clinical trials.^51–53^Fig. 1 depicts a schematic of chemiresistor, fabricated by spin coating the graphene nanocomposite sensing film on alumina substrate with electrodes and heater printed on the either side. The sensor was explored for acetone sensing properties in presence of other interfering biomarkers along with humidity in exhaled breath and used in clinical trials.^54^ The change in electrical properties of the sensors depends on the concentration of target biomarker. Various SMOs of different nanostructures have received a steady growth for their low cost fabrication process, reliable measuring electronics that demand very low power consumption.^55,56^ Indeed, the non-invasive sensing devices for clinical applications must possess satisfactory portability and affordable cost in addition to their demonstrated sensor performances. Hence it has spurred the interest of the international breath research community to develop an economical hand-held chemi-sensor that is capable of monitoring biomarkers in exhaled breath. The nanomaterials based chemi-resistive sensors' parameters such as sensitivity, selectivity, repeatability, response and recovery time can be optimized by altering the characteristics of the sensing material and their definition were given as follows:

*Relative response*: ratio of resistance of the sensor in the ambient atmosphere to the resistance to the exposure of acetone of varying concentration.^57^

1 *S* = (*R*_a_/*R*_g_) − 1

*Sensitivity*: change in relative response with respect to the change in gas concentration.^57^

*Selectivity*: measure of the relative response of other interfering biomarkers with respect to acetone.

*Repeatability*: measure of variation in relative response for the same gas concentration under the same experimental conditions.

*Stability*: measure of depreciation in sensor performance over a period of 1 month.

*Response and recovery time*: time taken by the sensor to reach 90% resistance change of the final equilibrium value.^58^

The basic sensing mechanism of these SMOs depends on the change in carrier concentration of the sensing material during gas interaction. This interaction may result in an increase or decrease in electrical resistance or conductivity.^59^ n-type SMOs resistance decreases for reducing gas and increase for oxidizing gas from the baseline resistance. Meanwhile, a *vice versa* phenomenon occurs for p-type SMOs, where resistance decrease for oxidizing gases and increase for reducing gases.^59^ Commonly, chemiresistors exhibit a change in its electrical resistance in the presence of specific target analyte it is engineered to, based on the following mechanisms.

### 2.1 Surface adsorbed oxygen ion mechanism

**Fig. 2:**
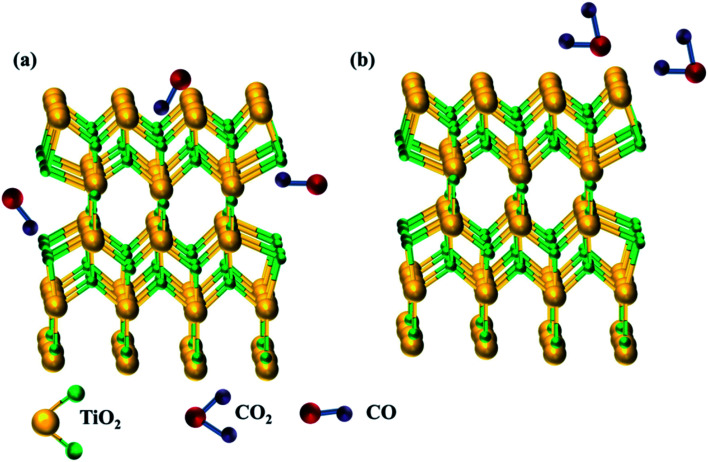
(a) Ionized oxygen ions adsorbed on the surface of the sensing film and (b) CO vapor interacts with ionized oxygen ions giving back electrons to SMOs.

Conventional SMO gas sensors exhibit a change in its electrical resistance by the redox reaction between the atmospheric oxygen ions and target analyte adsorbed on the surface of the sensing film. In an operating temperature between 200–500 °C, the atmospheric oxygen are ionized to O^−^, O^2−^ and O_2_^−^ by gaining electrons from the conduction band of the sensing film leading to oxidation, causing changes in its electrical resistance termed as baseline (Fig. 2a). The vicinity of oxidizing/reducing target vapor near the sensing film reacts with the ionized oxygen causing increase/decrease in its electrical resistance.^60^ For example, reducing carbon monoxide biomarker decreases the resistance from baseline (Fig. 2b) by contributing electrons as depicted in the equation,

2 CO_(gas)_ + O^−^_(ads)_ = CO_2(gas)_ + e^−^

In the presence of oxidizing NO_2_ biomarker, the resistance increases from the baseline as it gains electrons from the sensing film as depicted in the equation,

3 NO_2_ + e^−^ = NO^2−^

The basic challenge in such mechanism involves operating at higher temperature for physisorption and chemisorption of atmospheric oxygen. Also, the ionized oxygen species depends on the operating temperature, an important criteria for stabilizing the baseline. The molecular species of oxygen are high compared to atomic species at temperatures less than 150 °C.^61^ However, gas interaction occurs throughout the surface of the sensing material favorable for the transduction of whole concentration of target analyte at the vicinity of sensing material.^62,63^

### 2.2 Charge transfer mechanism

**Fig. 3:**
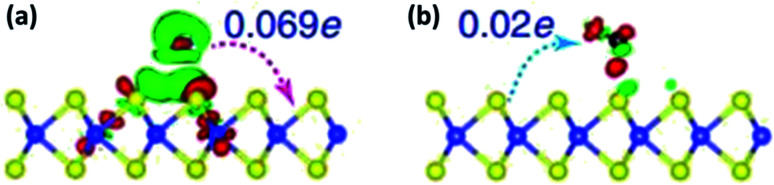
Difference in charge density and transfer process of (a) NH_3_ and (b) CO on monolayer MoS_2_ reproduced from ref. 65 with permission from Springer, Copyright 2021. The blue and yellow balls represent Mo and S atoms. The red and green charge distribution corresponds to charge accumulation and depletion.

The gas sensing mechanism of 2D layered nanomaterial such as graphene, MoS_2_*etc.* differs from conventional SMO sensors by charge transfer process. The electrical resistance changes due to the adsorption, charge transfer and desorption of target analyte on the sensing film. The material acts as either donor or acceptor, depending on the nature of the target analyte, leading to a different transfer direction and quantities of charges. Once re-exposed to air, desorption of target analyte leads the resistance to its baseline.^64^Fig. 3 shows the charge transfer and density difference between n-type monolayer MoS_2_ with a donor (NH_3_) and acceptor (CO), where green region represents charge accumulation and red region shows charge depletion regions. The free electrons in the donor vapor (NH_3_) donates electron onto the MoS_2_ monolayer leading to an increase in charge carrier thus decrease in resistance. On the contrary, charge transfer from the conduction band of the n-type MoS_2_ to the acceptor vapor (CO) causes the charge density to decrease and increase in electrical resistance of n-type MoS_2_.^65^ Unlike, the oxygen ion mechanism, physisorption dominates the adsorption of target analyte in two dimensional nanomaterials.^66^ Physisorption being a weak force and un-directional, strong and selective binding of analyte is hindered and lead to sluggish recovery, respectively.

### 2.3 Band bending mechanism

**Fig. 4:**
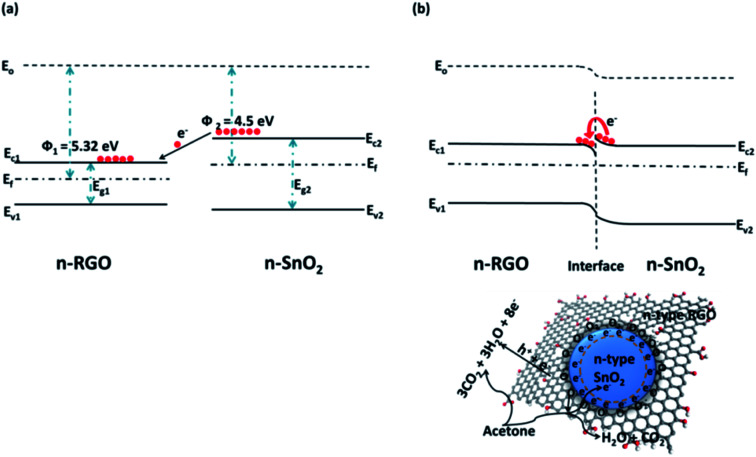
Energy band diagram of RGO/SnO_2_ (a) before and (b) after gas infusion reproduced from ref. 67 with permission from American Chemical Society, Copyright 2021.

Hybridization of SMOs with 2DMs creates heterojunction at the interface due the difference in their work function. Hence an obvious difference in Fermi energy levels causes an energy barrier height (Δ*E*) leading to accumulation and depletion region at the interface. The vicinity of target analyte near the heterojunction leads to the equalization of Fermi level due to band bending. Fig. 4 shows the energy band structure diagram of RGO/SnO_2_ before and after vapor infusion. Hybridization of SnO_2_ and RGO with a work function of 4.5 eV and 5.32 eV respectively causes a difference in Fermi energy levels (Fig. 6a). Further, infusion of donor vapor (acetone) leads to transfer of charge from lower work function (SnO_2_) to higher work function (RGO) tending to equalize the Fermi energy levels (Fig. 6b). This change in interfacial barrier causes a change in current flow characteristics across the rectifying heterojunction.^67^ The hybridization elucidates peculiar properties due to the synergistic effect between SMO and 2DMs. The band bending favors electron accumulation and chemisorption of donor species. The variation in band bending generates a more pronounced resistance variation which improves the gas sensing performance.^68^

Important milestones had been achieved in the way of non-invasive diseases diagnosis through portable breath analyzers by the discussed sensing mechanisms. Peng *et al.*^69^ had developed a device based on an array of chemiresistive nine functionalized gold nanoparticles to distinguish between healthy and lung cancer patients' breath. Recently, Blaikie *et al.*^70^ had demonstrated a compact device for the estimation of acetone concentrations under fasting, exercise and normal conditions based on cavity enhanced spectroscopic technique. Sun *et al.*^71^ had developed a transportable device to distinguish type-2 diabetic subjects from healthy subjects and validated with GC-MS with 600 breath samples. Gouma and team had demonstrated portable breath analyzers based on chemiresistive principle for various diseases (diabetes, renal diseases, lung diseases).^72–78^

**Fig. 5:**
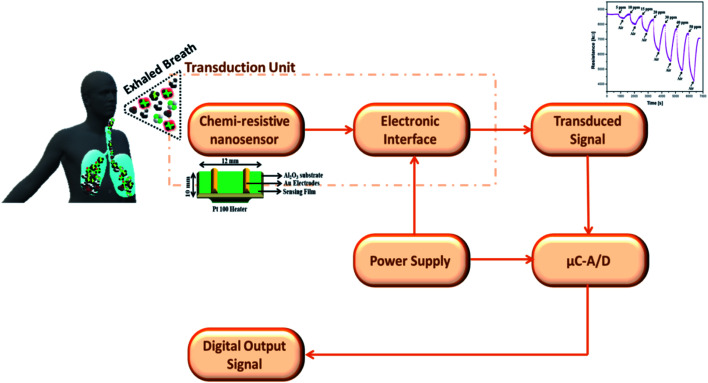
Block diagrammatic representation of chemi-resistive nanosensors in breath analysis application.

With the advancements of data acquisition instruments, the response to breath input from gas sensors can be digitized and transmitted to a computer for further signal preprocessing and feature extraction. Furthermore recent development of machine learning techniques provides better classification accuracy between healthy and diseased subjects. Hence, there is scope for improvement in each block represented in Fig. 5.

## 3. Nanomaterials as chemi-resistive gas sensors for breath analysis

VOCs exhaled from human breath provide information about the fundamental cellular mechanism which when detected quantitatively and qualitatively favors early detection of disease limiting further complications. This way of disease diagnosis had its footprint from the ancient times associating fruity like breath odor with diabetes mellitus, fishy with liver complications and urine like breath odor with renal failure. After the advent of modern equipment, approximately 30 VOCs with elevated concentrations in breath have been associated with halitosis, diabetes mellitus, kidney malfunction, asthma and different cancers. Over the past decades considerable efforts have been focused on nanostructured materials for the detection of various VOCs with better sensing performances. Materials at nanoscale possess peculiar property compared to their bulk counterpart. Exploiting these properties by controlling their size and shape in the form of nanorods, nanowires, nanosphere improved the gas sensing performance. Moreover, their working conditions including the operating temperature were found to significantly improve the detection capabilities selective to breath biomarkers.^79^ The motivation in the following section is to discuss these optimization strategies of promising nanomaterials as potential gas sensors incorporated in a breathalyzer for the detection of various disease biomarkers in exhaled breath.

### 3.1 Semiconducting metal oxide (SMO) nanostructures

Several different categories of material exhibit chemiresistive property. Among them, SMOs dominate as a superior gas sensing materials due to their capability to sense a wide variety of gases (oxidizing and reducing gases) and availability of numerous material choices.^80^ Moreover, as they are simple in operation and demands low power, SMOs were largely applied in gas and solvent leak detectors in semiconductor industries, environmental monitoring, food processing, agricultural industry and medical diagnosis.^81^ The first commercial SMO sensor at the beginning of 1970s used amorphous SnO_2_. Sensors made of SnO_2_ nanoparticles possess numerous advantages that include higher sensitivity, ability to operate at lower working temperature and thermally stable structure. This gas sensor was fabricated and patented by Taguchi which was later commercialized by Figaro Inc. The sensor was used for gas leak alarms in factories and residences to prevent fire accidents.^82^ The demand for high performance gas sensors for various applications with low power consumption had diverted the research efforts towards SMOs as gas sensors. Moreover, these SMOs can be altered to deliver high sensing performance by varying the concentration of dopant incorporated, controllable morphology, methodology of sensing material preparation and tailoring physical/chemical properties.^83–86^ However, sensitivity and selectivity being a primary sensing performance, various above mentioned optimization trials have been best possibly utilized.

**Fig. 6:**
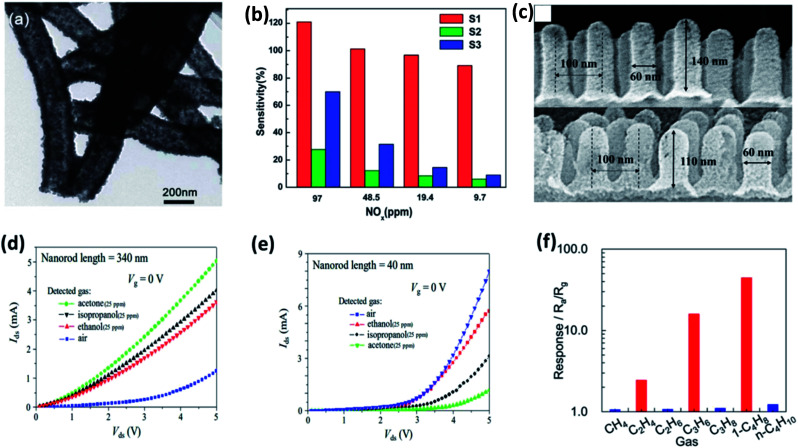
(a) TEM images of SnO_2_ hollow nanotube and (b) its gas sensitivities to NO_*x*_ with concentration ranging between 9.7 ppm to 97 ppm at room temperature reproduced from ref. 97 with permission from Royal Society of Chemistry, Copyright 2021. (c) SEM images of different dimensions of SnO_2_ nanorod array, their electrical characteristics in the presence of air and 25 ppm various disease biomarkers of (d) 340 nm and (e) 40 nm SnO_2_ nanorod array adapted from ref. 91 with permission from American Chemical Society, Copyrights 2021. (f) Selectivity plot of SnO_2_ nanosheets towards alkenes reproduced from ref. 92 with permission from American Chemical Society, Copyrights 2021.

The intriguing property of SMOs that it appears in diverse shapes (nano spear, nanowires, nanobelts and nanotubes) favors more interaction sites for target biomarkers. Thus, leading to better sensing performances by optimizing their morphology and tailoring its physico-chemical properties. The permeable and porous structures of metal oxide are explored beneficial for the efficient gas diffusion and entire electron depletion accordingly. The high sensing performances of metal oxides are defined by low detection limits and short recovery times.^87,88^ Numerous SMO nanostructures such as SnO_2_, WO_3_, In_2_O_3_, NiO, ZnO, CuO, Co_3_O_4_ has been utilized as standalone materials for the detection of various biomarkers.^89^ Jiang *et al.*, optimized 3 variations of the preparation procedure to synthesize SnO_2_ hollow nanotube observed from the TEM image shown in Fig. 6a. The detection capabilities of the prepared 1-dimensional hollow nanotube SnO_2_ were explored towards asthma biomarker (NO_*x*_). The sensors were capable of sensing NO_*x*_ at room temperature in the concentration ranging between 9.7 ppb to 97 ppm well below breath NO_*x*_ concentration with a faster response time of 20 s for 9.7 ppb and 6 s for 9.7 ppm (Fig. 6b). These sensing performances of the SnO_2_ nanostructured chemi-resistive sensors were attributed to its novel morphology favorable for gas interaction sites and the exposure of dominant crystal facets (101).^90^ The dependence of sensing performance on the morphology was further established by Xu *et al.*, where they studied the acetone, isopropanol and ethanol sensing properties of SnO_2_ nanorods at room temperature. They found the difference in sensing pattern with respect to the length of the synthesized nanorods shown in Fig. 6c–e.^91^ However, SnO_2_ nanosheets were found to be selective to ethane, propane and 1-butene compared to all other alkenes (Fig. 6f).^92^ Similarly, WO_3_ nanofiber exhibited a response of 4, whereas nanowire showed a response of 2 to 1 ppm acetone.^93^ These distinct sensing performances of the same nanomaterial with different morphology were ascribed to the modifications in grain boundaries, porous nature and surface area for interaction sites.

Concurrently, the peculiar strategy of modulating the operating temperature for selectivity tuning towards a specific VOCs were studied. A SMO sensor operating at different temperature may behave like a distinct sensor with different performance to specific VOC.^94–96^ SENSOR Lab at Bresica (Italy) has addressed the issue of selectivity tuning by modulating the operating temperature of ZnO nanoparticles. They found the sensor was selective to NO_2_ at 300 °C, H_2_ at 400 °C and CH_4_ at 500 °C and attributed to the thermodynamics of gas adsorption on the sensor surface including decomposition of gases on the surface.^97^ This strategy was also exhibited on the commercial sensors from Figaro Inc.^98,99^

Similarly, many other metal oxide nanostructure were utilized for sensing different biomarkers as listed in Table 2 by optimizing their morphology and operating temperature. The synergism between the optimization of morphology and operating temperature was explored in order to engineer the nanomaterial to selectively detect the biomarkers. Acetone was detected by ZnO nanomaterial with different morphologies such as flower shaped, brittle glass shaped and dandelion like spheres operated at 300 °C, 215 °C and 230 °C.^100,101,103^ Yet, ZnO nanosheets showed high sensitivity to formaldehyde and acetaldehyde with a lower detection limit of 50 ppb working at 220 °C.^102^ Likewise, In_2_O_3_ nanowires detected acetone at 400 °C, whereas thick wires detected low concentration (500 ppb) of NO_2_ at temperature less than 300 °C.^110^ Yet, In_2_O_3_ nanobricks detected 500 ppb of NO_2_ at a significantly lower operating temperature of 50 °C.^112^ These discussions illustrated the capability of tuning the selectivity by various optimization strategies. However, the response to exhaled concentration range tend to be poor due to lack in enhanced surface area for analyte interaction. In addition, meager conductivity of SMOs hinders the reproduction of the electrical change before and after analyte interaction to the outer world.

**Table 2:** The gas sensing performances of various SMOs with morphological optimization towards different diseases biomarkers in exhaled breath

Materials	Nanostructure	Gas	Disease biomarker	Concentration	Sensor response	Ref.
ZnO	3D hierarchical flower	C_3_H_6_O	Diabetes	100 ppm	18.6	100
ZnO	Brittle grass	C_3_H_6_O	Diabetes	100 ppm	107	101
ZnO	Nanosheets	Formaldehyde	Lung cancer	1 ppm	75%	102
		Acetaldehyde			77%	
ZnO	Dandelion like spheres	C_3_H_6_O	Diabetes	100 ppm	33	103
SnO_2_	Nanotubes	NO_*x*_	Asthma	9.7 ppb	16.1	90
SnO_2_	Thin films	NH_3_	Renal failure	50 ppm	6.94	104
SnO_2_	Microcubes	Toluene	Lung cancer	1 ppm	2.4	105
		Benzene			1.5	
SnO_2_	Flower-like nanostructures	Toluene	Lung cancer	100 ppm	9.7	106
		Formaldehyde			9.5	
WO_3_	Nanocolumns	C_3_H_8_O		100 ppm	3	107
WO_3_	Nanoplate	NO_2_	Asthma	5 ppm	10	108
WO_3_	Nanowire	NH_3_	Renal failure	300 ppm	2.39	109
In_2_O_3_	Nanowires	C_3_H_6_O	Diabetes	25 ppm	—	110
In_2_O_3_	Nanocrystals	NO_*x*_	Asthma	970 ppb	1.9	111
In_2_O_3_	Nanobricks	NO_2_	Asthma	500 ppb	402	112
Co_3_O_4_	Nanocube	C_3_H_6_O	Diabetes	500 ppm	4.88	113
	Nanosphere				1.62	
Co_3_O_4_	Nanosheet	C_3_H_6_O	Diabetes	100 ppm	6.1	114
	Nanofiber				4.0	
	Nanorod				2.7	

### 3.2 2D nanomaterials

On the other hand, 2DMs has intriguing properties such as high surface area, high conductivity and high carrier mobility at room temperature with low noise that facilitates better response for target analytes. Exploiting these merits, graphene and its derivatives were the most explored carbon materials for gas sensor application in the past decade. The transparent and flexible characteristics of graphene were utilized for different configuration gas sensing devices.^115–118^ The defects induced by surface functionalization may influence the gas adsorption and desorption by the increase in number of reactive sites causing high sensitivity. Numerous review articles on graphene based gas sensors with a special focuses on its application in different fields, transduction principles, functionalization with metals and polymers were presented.^119–126^

**Fig. 7:**
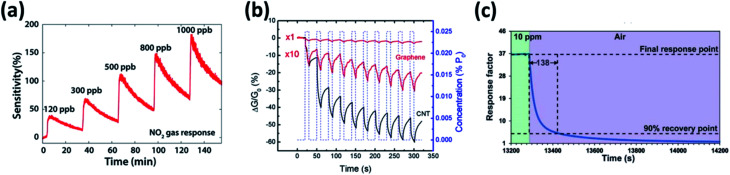
(a) Transient response of atomic-layered MoS_2_ to NO_2_ with concentration ranging from 120 ppb to 1000 ppb operated at room temperature reproduced from ref. 128 with permission from American Chemical Society, Copyrights 2021 (b) conductivity change of monolayer graphene upon exposure to 12 ppm TEA at room temperature reproduced from 129 with permission from American Chemical Society, Copyrights 2021 and (c) response/recovery of SnS_2_ to 10 ppm NO_2_ operated at 120 °C adopted from ref. 66 with permission from American Chemical Society, Copyrights 2021.

Inspired by the physic-chemical and electrical properties of graphene derivatives favorable for gas sensing applications, researchers are exploring over the periodic table for the search of ultrathin 2DMs. Among them, 2DMs represented by MX_2_, where M denotes the transition metals like Mo, W, Hf, Ti, Zr, V, Nb, Ta, Re, *etc.* and X denotes the chalcogens (Te, S or Se).^127^ However, only certain transition metal disulfides (TMDs) such as MoS_2_, WS_2_ and SnS_2_ have been utilized as a sensing film for the detection of various disease biomarkers in exhaled breath as listed in Table 3. In comparison with graphene (zero band gap), TMDs possess tunable bandgap (1–2 eV) with a shift from indirect to direct bandgap as a function of layer thickness due to exfoliation.^136^ Among other chalcogens, TMDs are widely explored due to its abundant presence in nature, less toxic and stable in atmosphere compared to metal selenides and metal tellurides. Their gas sensing performances were illustrated in Fig. 7. The NO_2_ sensing performance of atomic layered MoS_2_ nanosheet at low concentration of 120 ppb have not shown a complete recovery to the baseline and same occurrence was observed for subsequent concentrations (Fig. 7a).^128^ Identically, monolayer graphene exhibited no recovery to the baseline at 12 ppm triethylamine (TEA) as shown in Fig. 7b.^129^ Similar performance was witnessed by SnS_2_ nanosheets with a high response (∼170 s) and recovery time (∼140 s) to 10 ppm NO_2_ (Fig. 7c).^66^ This is attributed to their layered structure possessing weak van der Waals interlayer force favorable for exfoliation down to single and multilayers. However, their strong molecular interlayer force causes restacking of the layer.^137^ It is therefore desirable to composite them with a nanomaterial that acts as spacers which avoids restacking issues and facilitates porous structure for gas adsorption driven by high surface area, absorption coefficient and fast electron transfer.^138^

**Table 3:** The gas sensing performances of TMDs towards different diseases biomarkers in exhaled breath

Materials	Layer thickness	Gas	Disease biomarker	Concentration	Sensor response	Ref.
MoS_2_	Atomic	NO_2_	Asthma	120 ppb	35%	128
	1	TEA		1 ppm	3%	129
	Atomic	NH_3_	Renal failure	5 ppm	1%	130
	2	NO	Asthma	2 ppm	80%	131
WS_2_	5 nm	NO_2_	Asthma	5 ppm	68.4%	132
	110 nm	NH_3_	Renal failure	5 ppm	1.6%	133
	Thin film	NH_3_	Renal failure	5 ppm	0.2%	134
SnS_2_	1–3	NH_3_	Renal failure	100 ppm	2.13	135
		NO_2_	Asthma	10 ppm	36.33%	66

### 3.3 SMOs/2DM nanocomposites

The alternate approach of SMO/2DM binary nanocomposite may solve several challenges faced by standalone SMO or 2DM in enhancing the sensor performances in all the aspects. Their complementing features suitable for gas sensing applications are listed in Table 4. The synergism favors electronic, chemical and geometrical effects of the sensing material. The combination of SMOs and 2DMs may rectify the restacking problems of the multilayer 2D sheets and facilitates porous structure for enhanced gas diffusion. Further, the depletion layer formed at the interface of n–n, p–n or p–p heterojunction modulates due to gas diffusion and may enhance charge transport along with the favorable chemical bonds formed between SMO and 2DM.^139^

**Table 4:** Complementing features of SMOs and TMDs

	Advantages	Disadvantages
SMOs	Scalable fabrication	Slow carrier transfer
	Low cost	High operating temperature
	Long-term stability	Lower gas response
TMDs	Fast carrier transfer	Sluggish recovery
	Low operating temperature	Low selectivity
	High gas response	Lack of stability

As discussed in the previous section, SnO_2_ was widely used as a standalone material for gas sensors owing to its wide bandgap (3.6 eV). Moreover, its enhanced optical, electrical and electrochemical properties were suitable in various gas sensing device configurations. However, its capability of selectively detecting gas concentration as low as in parts per billion among diverse environment was hindered for real time breath analysis application. Hence, numerous efforts have been made to composite it with 2DMs for the detection of variety of breath biomarkers under varying structural morphology of the nanocomposite and working conditions of the sensor.^140^

**Fig. 8:**
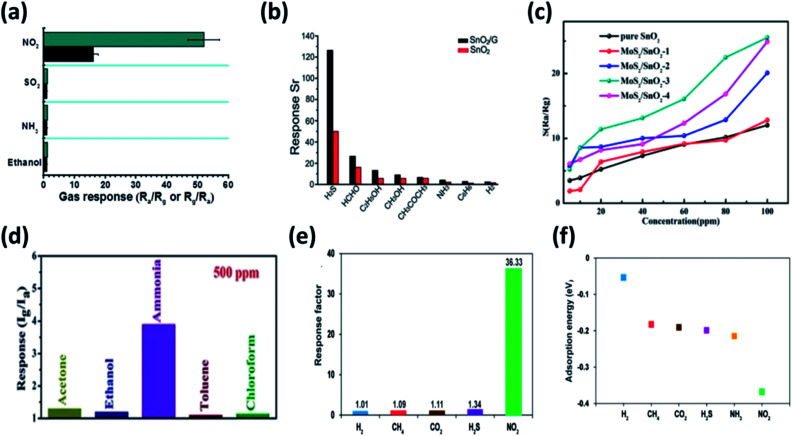
Selectivity of (a) graphene–SnO_2_ binary nanocomposite to 3 ppm NO_2_ at 150 °C reproduced from ref. 141 with permission from American Chemical Society, Copyrights 2021 and (b) graphene–SnO_2_ binary nanocomposite to 50 ppm H_2_S at 260 °C adapted from ref. 142 with permission from Royal Society of Chemistry, Copyrights 2021. (c) Response of different Mo/Sn molar concentration of MoS_2_/SnO_2_ sensor to varying concentration (5–100 ppm) of TEA at an operating temperature of 280 °C used from ref. 144 with permission from American Chemical Society, Copyrights 2021. (d) Selectivity characteristics of SnO_2_/SnS_2_ nanocomposite sensor to 500 ppm of ammonia and various gases at room temperature adapted from ref. 147 with permission from Royal Society of Chemistry, Copyrights 2021. (e) Response of SnS_2_ sensor to various biomarkers at 160 °C, (f) surface adsorption energy of SnS_2_ with studied biomarkers adapted from ref. 66 with permission from American Chemical Society, Copyrights 2021.

Graphene–SnO_2_ nanocomposite showed selective sensing to NO_2_ at 150 °C achieving a lower detection limit of 1 ppm with a response of 24.7 (Fig. 8a).^141^ Meanwhile, Zhang *et al.* has showed that graphene–SnO_2_ nanocomposite was also selective for H_2_S operated at 260 °C with a detection limit of 1 ppm (Fig. 8b).^142^ Further, SnO_2_ with various graphene derivatives was found to exhibit superior sensing performances detailed in our previous review.^143^ Besides, composition of SnO_2_ with TMDs may elucidate peculiar properties due to synergism between them. The most widely used MoS_2_/SnO_2_ p–n heterojunction among other TMDs, demonstrated better sensing performances to TEA at an operating temperature of 230 °C (Fig. 8c). The performance of Mo/Sn ratio of 0.53 dominated other mole ratios of the composite. The sensor showed a response of 24.9 for 100 ppm of TEA with a detection limit of 5 ppm.^144^ Meanwhile Han *et al.*, investigated the detection of NO_2_ at room temperature by varying the Mo/Sn molar ratio. The study revealed that 1.25 mL of stannic chloride with 20 mL MoS_2_ nanosheet dispersion (MS-1.25) showed highest response of 18.7 to 5 ppm NO_2_. Moreover the response time, complete recovery, long term stability and selectivity of the composite was significantly improved. The authors speculated the enlargement of surface area by the incorporation of SnO_2_ on MoS_2_ nanosheets and the p–n heterojunction changed the electronic properties locally.^145^ The humidity sensing property of WS_2_/SnO_2_ revealed the importance of 2DM/SMO composites, where a significant improvement in sensing performances (862.8 times) were observed compared to standalone SMO or 2DM.^146^ Moreover, with much electronegative in nature, SnS_2_ was made composite with SnO_2_ to improve the selectivity towards ammonia and NO_2_.^147^ The study by Li *et al.* showed significant selectivity to ammonia with a response of 2.48 for 100 ppm at room temperature among other breath biomarkers (Fig. 8d). The sensor exhibited linear response at exhaled breath concentration range.^147^ However, Ou *et al.*, has established a strong detection of NO_2_ by SnS_2_ at lower operating temperature of 160 °C compared to other VOCs (Fig. 8e) and capable of detecting as low concentration as 0.6 ppm. They found the high selectivity of NO_2_ at 160 °C due to the lower adsorption energy of NO_2_ to SnS_2_ as shown in Fig. 8f.^66^

**Fig. 9:**
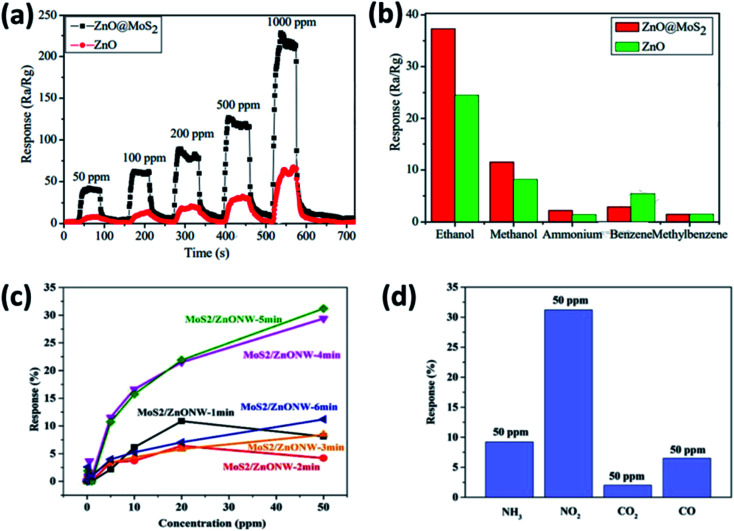
(a) Typical response of ZnO@MoS_2_ to ethanol at concentration ranging between 50 ppm to 1000 ppm operated at 260 °C, (b) selectivity characteristics of ZnO@MoS_2_ to 100 ppm ethanol at an operating temperature of 260 °C adopted from ref. 157 with permission from Elsevier, Copyrights 2021, (c) response of different sputtering time MoS_2_ on ZnO nanowires to varying concentrations of NO_2_ at 200 °C and (d) selectivity characteristics of MoS_2_/ZnO to 50 ppm NO_2_ at an operating temperature of 260 °C reproduced from ref. 156 with permission from Elsevier, Copyrights 2021.

Similarly, many other combinations of SMO/2DM were exploited for the detection of various exhaled biomarkers listed in Table 5 by various optimization strategies. Yan *et al.*, has investigated the sensing performance of ZnO coated MoS_2_ to ethanol concentration ranging between 50–1000 ppm at an operating temperature of 260 °C. Their results suggested the remarkable sensing behavior of ZnO@MoS_2_ binary metal oxide nanocomposite compared to pure ZnO in terms of sensitivity and selectivity to ethanol (Fig. 9a and b). These results are attributed to the development of interface at the heterojunction leading to rapid electron transfer by the direct conduction paths provided by MoS_2_.^157^ Following this finding, Zhao *et al.*, optimized the layer thickness of MoS_2_ on ZnO nanowires by varying the sputtering time to investigate NO_2_ sensing behavior at 200 °C. The sensor exhibited excellent sensitivity with a detection limit of 200 ppb favorable for breath analysis (Fig. 9c). Furthermore the sensor showed good selectivity to 50 ppm of NO_2_ in comparison with the same concentration of NH_3_, CO_2_ and CO along with stable recovery and repeatability (Fig. 9d).^156^ Meanwhile, Qin *et al.* has hybridized TiO_2_ with WS_2_ with different molar ratio (0.44 to 1.65) to improve the selectivity of WS_2_ 2DM towards ammonia at room temperature. The composite with a molar ratio of 0.44 TiO_2_ QD/WS_2_ exhibited fast response and high selectivity to 500 ppm ammonia.^163^ Furthermore, tungsten was also used in combination with WS_2_ to detect ammonia, H_2_ and NO_2_. They optimized the working temperature at 150 °C and achieved a lower detection limit of 1 ppm to ammonia.^168^ These discussions provide a notion that different combination of SMO/2DMs with varying optimization strategies such as morphology, nanosheet's layer thickness and operating temperature would tune the sensing system towards specific biomarker with enhanced sensitivity. Similarly many other binary and ternary nanocomposites with 2DMs can be explored for elucidating the peculiar properties and explore them for the detection of various breath biomarkers. Incorporation of these sensors in handheld portable breath analyzer may possibly favor the underprivileged community in the poor healthcare resource setting. The biomarker concentration displayed on the system or rather transmitted wirelessly could pave way for universal use, for its effortless sample collection procedures. In the future, SMO/2DM based nanosensors could be miniaturized on a single lab-on-chip and could be used as a pocket device or an add-on to the mobile phones.

**Table 5:** The gas sensing performances of various SMO/TMD nanocomposites towards different diseases biomarkers in exhaled breath

SMO	2DM	Gas	Disease	Concentration	Response	Ref.
SnO_2_	Graphene	NO_2_	Asthma	5 ppm	72.6 (*R*_g_/*R*_a_)	141
		H_2_S	Halitosis	50 ppm	130 (*R*_a_/*R*_g_)	142
	Reduced graphene oxide	NH_3_	Renal failure	1000 ppm	—	148
		HCHO	Lung cancer	10 ppm	435	149
	MoS_2_	TEA		100 ppm	24.9 (*R*_a_/*R*_g_)	144
		NO_2_	Asthma	5 ppm	18.7 (*G*_g_/*G*_a_)	145
		CH_4_		100 ppm	2.014	150
		NH_3_	Renal failure	50 ppm	10%	151
	WS_2_	Humidity		95% RH	8.5 (*R*_a_/*R*_g_)	152
	SnS_2_	NH_3_	Renal failure	100 ppm	2.48 (*I*_g_/*I*_a_)	147
		NO_2_	Asthma	10 ppm	36.33	66
ZnO	Reduced graphene oxide	NO_2_	Asthma	0.5 ppm	12 (*R*_g_/*R*_a_)	153
	MoS_2_	C_3_H_6_O	Diabetes	5 ppm	14.40 (*R*_a_/*R*_g_)	154
		NH_3_	Renal failure	100 ppm	61.92%	155
		NO_2_	Asthma	50 ppm	31.2%	156
		C_2_H_5_OH	Alcohol consumption	50 ppm	42.8 (*R*_a_/*R*_g_)	157
WO_3_	Graphene	H_2_S	Halitosis	5 ppm	65.5 (*R*_a_/*R*_g_)	158
		C_3_H_6_O	Diabetes	5 ppm	13.7 (*R*_a_/*R*_g_)	
		NO_2_	Asthma	5 ppm	133 (*R*_g_/*R*_a_)	159
	MoS_2_	H_2_S	Halitosis	25 ppm	20%	160
TiO_2_	Reduced graphene oxide	Methanol		800 ppm	96.3%	161
	MoS_2_	C_2_H_5_OH	Alcohol consumption	100 ppm	14.2	162
	WS_2_	NH_3_	Renal failure	500 ppm	56.69	163
Fe_2_O_3_	Reduced graphene oxide	H_2_S	Halitosis	1 ppm	9.2	164
Co_3_O_4_	Graphene oxide	C_3_H_6_O	Diabetes	5	2.29 (*R*_g_/*R*_a_)	165
	MoS_2_	NH_3_	Renal failure	0.1	10.3%	166
Cu_2_O	Graphene	H_2_S	Halitosis	100 ppb	36%	167

## 4. Socio-economic demands and companies

Nanosensors based on chemi-resistive principle for breath analysis has seen progress in recent years in terms of sensitive and selective detection of specific breath biomarkers amidst various interference including exogenous gases and humidity in breath.^169–171^ Meanwhile with low power demand resulting in the reduction of device dimension on a single lab-on-chip, nanosensors are the choice of technology for incorporation in portable breathalyzers for disease diagnosis. Also, an array of cross-reactive nanosensors incorporated in the breathalyzer would mimic the mammalian olfactory and detect various gases emanating from breath probing the metabolic processes at the fundamental cellular levels favoring early detection of diseases. Accurate detection of these trace level gases would pave way for non-invasive, user friendly, personalized home care diagnostic equipment which lead to better control of diseases and monitor the effect of medication. Moreover, the instant display of results may avoid pre-analytical and post-analytical processes such as sample collection, preparation and test report dispatch. Also it enables mass screening during pandemic leading to reduced transmission rate of the disease.^172^ These processes eliminate consumables thus considerably reduce the global health expenditure. The merits of nanosensors create a demand for breathalyzers in global market demanding a socio-economic balance. Hence researchers across the globe are having high hopes and making strides on startups for disease diagnosis through breath analysis by various technologies. For instance, SpiroNose® developed by Breathomix utilizes 7 different SMO sensors that capture real-time breath profiles. The between-days repeatability of asthmatic and healthy subjects was precise and was even better when compared to the routine standard test (spirometry).^173^ Moreover, it satisfactorily discriminated healthy subjects and patients with COPD, asthma and lung cancer.^174^ Similarly, Aeonose® developed by The Enose Company, Netherlands have been found suitable for the prescreening of prostate, lung and collateral cancer proving it suitability for personalized home care.^175–179^ Also, AmBeR® was designed and developed by BreathDX consists of an array of sensors fabricated by inkjet printing functional nanomaterials capable of ammonia measurement in ppb concentration range.^180,181^ Each of the startup listed in Table 6 is focused on identifying various disease by fabricating a sensor selective to specific biomarkers and using other analytical technologies.

**Table 6:** List of startups across the globe focusing on various disease diagnoses through breath analysis involving different technologies

Sl. no.	Company	Country	Inception	Focus	Product & technology
1	Breathomix	Netherlands	June 2018	Cancer, inflammatory and infectious diseases	SpiroNose® – 7 different MOS sensors
2	The Enose Company	Netherlands	2013	Oncology, infectious-, and neurological diseases, colon cancer, lung cancer and tuberculosis	Aeonose® – metal oxide semiconductors
3	Owlstone Medical	UK	2005	Liver disease, colon cancer and lung cancer	Field Asymmetric Ion Mobility Spectrometry (FAIMS)®
4	Breath Diagnostics Inc.	US	2014	Lung cancer	OneBreath® – mass spectrometry
5	BreathDX	UK	—	Ammonia quantification	AmBeR® – nanosensors
6	CAIRE Diagnostics formerly Spirosure Inc.	US	2012	Allergic airway inflammation, asthma and other pulmonary conditions	Fenom Pro® – electrochemical
7	Breath Analyzers Pte. Ltd.	Singapore	2016	Gastrointestinal diseases, helicobacteriosis	HepyScreen®
8	Algernon Pharmaceuticals formerly Breathtec Biomedical Inc.	Canada	2015	Liver disease – Non-Alcoholic Steato Hepatitis (NASH), chronic kidney disease and inflammatory bowel disease	—
9	New England Breath Technologies	UK	2018	Type 2 diabetics	Glucair®
10	Deep Breath Initiatives	Switzerland	2018	Therapeutic drug monitoring	Mass spectrometry

## 5. Challenges and regulatory aspects

Even though with the high socio-economic demand, inception of startups, research and development across the globe with the state-of-the-art technologies, there are numerous challenges needs to be addressed. For the commercial deployment of breathalyzer for non-invasive disease diagnosis in clinical setup and for personalized homecare utilization, much care must be provided in designing a nanomaterial based chemi-resistive sensor. Sensors should possess accurate detection capabilities as the breath sample is highly complex and informative due to the changes in breath volatolomics that occurs on the fundamental cellular processes. Moreover, the biomarker is in parts per million which demands the sensor to possess high sensitivity and selective to detect the trace level amount among other interfering biomarkers along with the strong influence of relative humidity in exhaled breath. Thus the sensor incorporated in breathalyzer should possess superior gas sensing performances such as sensitivity and selectivity towards specific disease biomarkers, humidity-resistive property, faster response and recovery time *etc.*

However, desiccants and pre-concentrators are crucially important, if the sensors do not satisfy the humidity-resistive property and selectivity, respectively.^182^ Moreover, as only the alveolar breath contains information about the cellular processes, the sensor chamber should be designed in such a way to accommodate the whole of alveolar breath and avoid sharp edges to eliminate recirculation zones. There is still no standardization on the collection of sample from either oral or nasal cavity. Hence, influence of exogenous production and presence of the biomarker as a pollutant in the atmosphere may hinder the exact quantification of endogenous production of biomarker.

More importantly, the biomarkers associated with specific diseases by the analytical techniques such as GC-MS is questionable as the pathophysiology of a single disease may diffuse numerous VOCs. Therefore, a global library of VOCs with corresponding diseases needs establishment to develop a cloud of breath database which may act as a reference standard. Further, the measuring electronics requires proper optimization to avoid instrumentation errors after proper breath sample collection and sensor design. The lack of standardization of library of VOCs, breath sample collection procedure, chamber design, sensor positioning and measuring electronics causes an insignificant criterion for reproducibility of the diagnostic outcomes.^183,184^ The mentioned issues in standardization lead to huge variations in results between different studies and are hard to replicate.^185^ Further, the sensors work in harsh environment (humidity and temperature) leading to eventual degradation in sensor performance. Hence, as there are opportunities and demand for a breathalyzer, corresponding challenges and improvements are necessary.

The use of nanomaterials for medical devices and implant materials has been successfully explored. Particularly in USA and Taiwan, nanomaterial incorporated products were widely seen that exhibits better performance compared to their bulk counterpart. However, safety and security being a primary concern, the considerable change in properties of the nanomaterial used in a medical device requires detailed study on their adverse effects on human body including toxicity. The dimensions of the nanoparticle decide its hazardous nature. Smaller particles may enter the human body through respiratory tract during inhalation, skin or oral ingestion due to the poor immune system of human body towards nanoparticle. For instance, nanoparticles less than 30 nm in size may damage the central nervous system, the defective mechanism of lung cells is not capable of handling nanoparticle with the size lesser than 70 nm and nanoparticle size less than 50 nm may enter the nucleus of human cells.^186,187^

Hence, nanomaterial based medical devices have raised the concern for health authorities to develop certain regulatory aspects for safety assessment that does not develop any temporary or permanent discomfort to the users. The risks associated with the absorption or distribution of nanomaterial in human body must be established. Along with these safety assessments, no compromise in the quality must be ensured by the manufacturer by focusing on repeated validation on the manufactured nano medical device.^188^ Hence, regulatory requirements are good manufacturing procedures, labeling and warning, approval procedures and post market follow up.

## 6. Conclusion and future outlooks

Non-invasive disease diagnosis through breath analysis is one of the foreseen healthcare services. Their tendency to replicate the fundamental cellular process makes it more attractive for early diagnosis leading to the increases in survival by adopting effective treatment methodologies. To this direction, chemi-resistive VOC sensors with a well understood sensing mechanisms for simple, fast and accurate detection of breath biomarkers were presented in this current review. These chemi-resistive gas sensors possess miniaturization capabilities with no trade-off on the performance, which pose as a primary requirement for point-of-care medical devices. As mentioned in this review, numerous SMO nanostructures, 2D nanosheets and their composites have been utilized for the detection of various disease biomarkers. Their demonstrated performances were favorable for diagnosis of disease from exhaled breath with an easy sampling procedure and instant display of the outcome. Besides, their mass-screening competency during COVID-19 pandemic has found a new market for portable breathalyzers.

Whilst, the strength of the technology remains weak and inconclusive. This reflects the lack of standardization of breath sampling, sensor technologies and sample sizes across the spectrum of studies. Under-powering alongside difficulties including inference by numerous other external sources of VOCs and complexity of breath have both contributed to the setback of the technology for regular clinical trial. It seems that the implementation of gas sensors for breath analysis application has not reached the maturity for the widespread acceptance and usage. Nonetheless, SMO/2DM nanocomposites are amongst the few materials that enhance geometrical, chemical and electronic properties for gas sensing applications to tune its selective binding with a single biomarker at lower concentration. Further, placement of cross reactive sensors and coupling of breath sample to sensors through pre-concentrator devices on a single lab-on-chip may realize the dream of non-invasive disease diagnosis through breath analysis.

However, with the rapid advancements in the field of nanotechnology, regulatory aspect of these medical devices essentially takes care of all the relevant issues. The quality and safety of these devices must be enforced by the regulatory authorities in order to protect the manufacturing staffs and the end-users. Meanwhile, frequent updates are necessary with respect to the emerging applicability of nanomaterials and devices.

## Conflicts of interest

The authors declare no conflict of interest, financial or otherwise.

## Supplementary Material
